# Geminiviruses and Food Security: A Molecular Genetics Perspective for Sustainable Agriculture in Africa

**DOI:** 10.3390/plants13192768

**Published:** 2024-10-02

**Authors:** Minenhle Felicia Zenda, Priscilla Masamba, Farhahna Allie, Abidemi Paul Kappo

**Affiliations:** Department of Biochemistry, Auckland Park Kingsway Campus, University of Johannesburg, P.O. Box 524, Johannesburg 2006, South Africa

**Keywords:** Geminiviruses, food security, genome editing, agriculture, Africa, crop improvement

## Abstract

The African continent is vulnerable to food insecurity. Increased food costs, job losses, and climate change force Africans to chronic hunger. Biotechnology can be used to mitigate this by using techniques such as CRISPR/Cas9 systems, TALENs, and ZFNs. Biotechnology can utilize geminiviruses to deliver the necessary reagents for precise genome alteration. Additionally, plants infected with geminiviruses can withstand harsher weather conditions such as drought. Therefore, this article discusses geminivirus replication and its use as beneficial plant DNA viruses. It focuses explicitly on genome editing to increase plant resistance by manipulating plants’ salicylic acid and jasmonic acid pathways.

## 1. Introduction

Africa is regarded as a poor continent. It has a low gross domestic product per capita, increased hunger, and food insecurity as after-effects of COVID-19. The coronavirus pandemic has caused a regress in African countries attempting to fulfill the Sustainable Development Goals (SDGs) by 2030. A 2-year rampant COVID-19 pandemic eliminated more than four years of progress in eradicating poverty, increasing the number of people living in poverty [1]. Poverty is a daily event in which a person in a community cannot access financial resources to provide essential needs for a minimum standard of living. This includes improper housing, lack of medical care, and access to clean water and healthy food [2]. In 2020, 490 million Africans lived in poverty, and 281.6 million were undernourished [3]. The prolonged lack of healthy food and clean water leads to chronic hunger. More so, extreme poverty in sub-Saharan Africa (SSA) has caused chronic need and increased vulnerability of the region to food insecurity. A person is regarded as food insecure when one cannot have physical and financial access to enough safe and nutritious food for proper growth and development to lead an active and healthy life [1,4,5]. Africa has the highest percentage of people experiencing poverty in the world, who do not have access to nutritious food or are unable to generate it for themselves. Since 1990, SSA has been regarded as food insecure. In 2020, the SSA region had the highest population of food-insecure people, with 239 million reported [6].

The COVID-19 pandemic worsened the complexity of food insecurity and elimination of hunger, poor international response, food scarcity, poor agricultural production, and poor storage techniques. In most African countries, food insecurity is formed by poor distribution, poor agricultural policy, infestation of pests and diseases, and climate change [3]. Climate change has a significant impact on crop production. The tripartite effects of increased temperature, water variability, and increased occurrence of extreme weather (droughts and floods) have unfavorable impacts on plants. Severe weather conditions, especially drought, are the most significant causes of food insecurity. The multi-year drought in the “Horn of Africa” has resulted in more than 36 million people being food insecure, and this is expected to continue in 2024. Climate change influences an increase in pest populations, thus causing an increase in plant diseases affecting agriculturally essential foods such as grains, legumes, cassava, and tomatoes. However, the combination of harsh weather conditions and viral plant diseases weakens the plant’s tolerance to abiotic stressors. For example, tomato plants diseased with the Tomato Yellow Leaf Curl Virus (TYLCV; Genus: *Geminiviridae*) have decreased heat tolerance [7].

Geminiviruse*s* are a large group of plant viruses that are amongst the most dangerous viruses that affect plants; they contribute to a significant decrease in global agricultural output. Geminiviruses caused a loss of more than USD 300 million in the Indian bean industry, up to 100% yield loss of tomato crops in Italy and the Dominican Republic, and a loss of almost USD 2 billion in African cassava production [8,9,10]. Geminiviral endemics are associated with an unusually high insect vector population [11,12]. Geminiviruses are small viruses with circular, single-stranded (ss) DNA. Therefore, they are ideal for DNA replication studies and are used in plant biotechnology for gene editing. RNA viruses such as the potato virus X (PVX) and tobacco mosaic virus (TMV) have been used in gene modification studies [13,14]. However, there is growing interest in using ssDNA viruses. Geminiviruses are being used for gene modification using zinc finger nucleases (ZFNs), transcription activator-like effector nucleases (TALENs), and clustered regularly interspaced short palindromic repeats (CRISPR)/Cas systems [15]. Geminivirus replicons (GVR) are adapted for gene manipulation to study gene function, insertion/knockout of genes of interest, and increase plant resistance [16,17,18]. This article will discuss geminivirus replication, the use of GVRs in plant biotechnology, and tolerance to abiotic stress induced in plants by geminivirus infection.

## 2. Geminiviru*s* Replication

Geminiviru*s* is a plant virus family that includes many DNA viruses that affect agriculturally and economically essential vegetables, including medicinal and aromatic plants. They can infect both monocotyledonous and dicotyledonous plants. Common symptoms associated with their infection are leaf curling, vein yellowing, yellow mosaic patterns, leaf dwarfing, and decreased fruit sets [19,20,21]. Severe illness could cause up to 100% yield loss. They are transmitted by four hemipteran families, *Aphididae* (aphids), *Cicadellidae* (leafhoppers), *Membracidae* (treehoppers), and *Aleyrodidae* (whiteflies) [22,23], and are classified according to the type of insect vector, genomic organization, host range, and phylogenetic reconstruction [24]. Their ssDNA is non-enveloped, and encapsulated in an elongated twinned-icosahedral capsid. This virus family has more than 500 species classified into 14 genera, namely, *Becurtovirus* (3 members), *Begomovirus* (445 species), *Capulavirus* (4 members), *Citlodavirus* (4 species), *Curtovirus* (3 species), *Eragovirus* (1 member), *Grabloviru*s (3 members), *Maldovirus* (3 species), *Mastrevirus* (45 species), *Mulcrilevirus* (2 species), *Opunovirus* (1 member), *Topilevirus* (2 members), *Topocuirus* (1 member), and *Turncurtovirus* (3 species).

**Figure 1:**
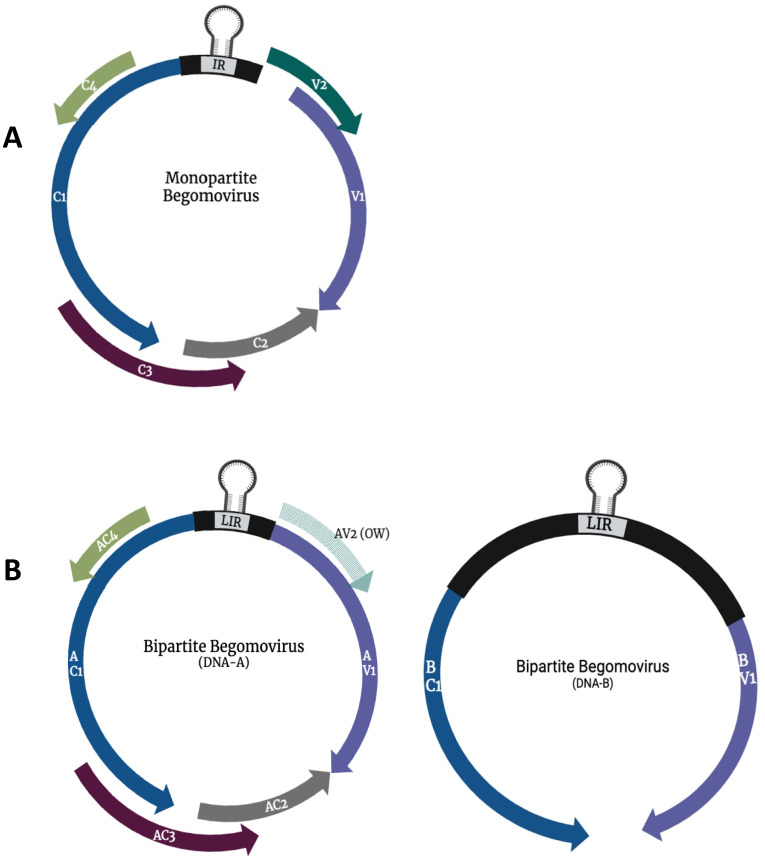
Genome organization of the monopartite (**A**) and bipartite (**B**) members of the genus Begomovirus. Arrows represent open reading frames. V1/(AV1) coat protein gene, V2/AV2 precoat protein gene, C1 (AC1) replication initiator protein gene, C2 (AC2) transcription activator protein gene, C3 (AC3) replication enhancer protein gene, C4 (AC4) symptom determinant protein gene, BV1 nuclear shuttle protein gene, and BC1 movement protein gene.

Begomoviruses are the largest group within the virus family. They are exclusively transmitted by the B-type whitefly *(Bemisia tabaci*) in a circulative persistent manner [25]. The genus includes plant pathogens that cause severe threats to significant crops, such as African Cassava Mosaic Virus (ACMV), TYLCV, and Tomato Curly Stunt Virus (ToCSV). Begomoviruses either have a monopartite, containing a single genomic (DNA-A) component, or bipartite, containing two genomic (DNA-A and DNA-B) components [26,27,28]. The genome size of begomoviruses ranges between 2.5 and 3.2 kb each, depending on their genome organization, with bipartite having a total of up to 5.2 kb. The begomovirus genome has up to eight overlapping open reading frames (ORFs) (Figure 1). However, these viruses only encode one structural protein, capsid protein (V1/AV1) [29]. Begomovirus bidirectionally transcribes their complementary and virion-sense strands. The viruses encode up to four proteins that are associated with virus replication in their complementary sense: replication initiator protein (C1/AC1/Rep), transcription activator protein (C2/AC2/TrAP), replication enhancer protein (C3/AC3/REn), and symptom determinant protein (C4/AC4) [30]. Their virion-sense strands encode two proteins responsible for encapsidation and movement: coat protein (V1/AV1/CP) and movement protein (V2/AV2/MP). The DNA-B of bipartite geminiviruses encodes for two proteins, nuclear shuttle protein (BV1) and movement protein (MP/BC1), that facilitate the intra- and inter-cellular movement of the virus (Figure 1).

All geminiviruses have an intergenic region (IR) where coding regions of both the virion and the complementary sense diverge outward. In some instances, some genera have a large IR (LIR) that is similar in size to that of the basic IR and a small IR (SIR) [31]. IR and LIR contain a common region (CR) region of approximately 200 nucleotides and do not encode any proteins [32]. The IR and LIR have a conserved origin of replication (*ori*) consisting of the invariant nano-nucleotide sequence ‘TAATATTAC’. However, the nano-nucleotide sequence for *Becurtovirus* and *Eragrovirus* slightly differs from that of a nucleotide sequence ‘TAAGATTCC’ [19].

The geminiviru*s* genome does not encode any accessory machinery needed for replication. Therefore, replication occurs in the virus-host plant nucleus. He and colleagues [24] recently found that replication occurs in the insect vector. TYLCV amplifies within the primary salivary glands of the whitefly, and it recruits the proliferating cell nuclear antigen (PCNA) and DNA polymerase δ enzyme. Whole virus particles are essential for successful transmission and infection. Transmission occurs in three stages involving acquisition, retention, and ejection of the virions through to the hosts’ phloem sap [33]. These viruses have developed strategies to enable successful replication inside the insect vector and host plant. Replication of the geminiviruses occurs in the nucleus of infected plant cells using recombination-dependent replication and rolling circle replication (RCR) [34,35].

During RCR, the viral ssDNA is uncoated upon entering the plant cell, forming a complex with the CP. This allows for the entry into the nucleus facilitated by nuclear localization sequences with the CP [36,37]. There is no direct implication of the CP in viral replication. However, mutation of the TYLCV- and ToLCV-CP causes a decrease in viral ssDNA [38,39]. The ssDNA is converted into dsDNA (dsDNA replicative form). The initial ssDNA to double-stranded (ds) DNA synthesis mechanism remains unknown. However, DNA polymerase α has been suggested to be essential for the generations of the dsDNA replicative form [40]. The dsDNA replicative form serves as a template for the transcription and replication of the viral genes. The Rep is the only protein that is essential for replication. The REn and other host accessory replication machinery enhance replication efficiency. The Rep oligomers are complex and interact with different replication factors to create a favorable environment [41,42,43,44]. Furthermore, it attracts the necessary host machinery required for replication. The Rep interacts with (1) plant retinoblastoma-related protein (RBR), thus inhibiting the interaction between the RBR and E2F, resulting in the freeing of the activator class of DNA replication machinery such as the PCNA, (2) small ubiquitin-related modifier (SUMO)-conjugating enzyme (SCE1) to presumably alter the SUMOlylation level of targets, thereby creating a stable environment for viral DNA replication [45,46].

The multifunctional Rep protein can confer virus-specific recognition of its cognate origin of replication, initiate (+) strand DNA replication, regulate its transcription, and enhance some geminiviruses’ transcription of late genes [47,48,49,50,51]. Early investigations of the dependence of geminivirus genome amplification on the Rep protein using the tomato golden mosaic virus (TGMV) by Elmer and colleagues [52] showed that mutations on the Rep protein result in the total abolishment of TGMV replication. The Rep protein will detect the conserved repeating motif in its (+) strand origin and cleave single-stranded DNA in the invariant sequence of the hairpin loop, indicative of its endonuclease activity, initiating RCR. The conserved stem-loop cleaves between the seventh and eighth nanomer sequence (TAATATT↓AC) [48]. The Rep protein functions as a helicase to unwind the dsDNA, creating a site conducive to constructing a Rep replication complex. The Rep complex includes replication machinery such as Replication protein A (RPA), PCNA, and Replication factor C (RFC). This replication complex assumes the role of a replisome.

**Figure 2:**
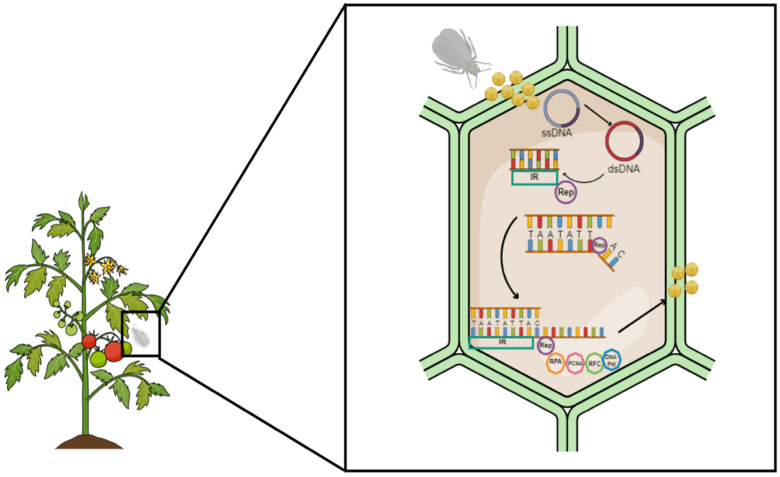
Begomovirus rolling circle replication. Viral particles are transmitted into the host by insect vector feeding. The ssDNA is converted to a dsDNA by an unknown mechanism. The Rep oligomer (purple) binds to the intergenic region (IR), unwinds the dsDNA, and cleaves at the *ori.* The Rep recruits various host replication factors, such as RPA (orange), PCNA (pink), RFC (green), and DNA polymerase (blue). A new strand of DNA is generated from the 3′-end. Once the mononucleotide is generated, the new ssDNA is cleaved and ligated to produce encapsulated progeny viral DNA.

During viral replication, the RPA increases the ATPase activity of the Rep protein [53]. The function of the RPA in plant DNA replication is to bind to ssDNA to protect it and prevent it from unfolding. It also coordinates the assembly and disassembly of host factors needed during replication. The mung bean yellow mosaic India virus (MYMIV) Rep-RPA complex extensively unwinds the origin of replication, thus facilitating the development and advancement of the replication fork. The Rep-RPA complex will then recruit the sliding clamp, loading RFC to aid in loading PCNA using ATP as an energy source. PCNA will circulate the Rep-RPA-RFC complex on the free 3-OH following DNA polymerase α (Pol α) to assist in viral amplification. Wu and colleagues [40] suggested that the Pol α is essential for the initial synthesis of the viral complementary strand and, therefore, for generating the dsDNA replicative intermediate, ultimately limiting the accumulation of both dsDNA and ssDNA. Another DNA polymerase enzyme implicated in viral DNA replication is DNA polymerase δ (Pol δ), which interacts with Rep and REn proteins. The viral REn is associated with the Rep complex to increase replication efficiency. A lack of the REn results in a decrease in the accumulation of both dsDNA and ssDNA of viral DNA. When a new viral DNA strand has been synthesized, the Rep will disassociate it from the dsDNA replicative fork by cleaving. The Rep will ligate the new ssDNA strand together to form an ssDNA progeny that will be encapsulated and transported out of the nucleus to the distal parts of the plants for systemic infection (Figure 2).

## 3. Secondary Effects of Geminivirus Infection (Particularly TYLCV)

Plants are affected by biotic stressors, and abiotic factors like heat, salinity, and drought can also negatively affect plant growth. Thus, prolonged exposure to such environmental conditions results in an overall reduction in vegetation yield. The International Food Policy Research Institute [54] has predicted that by 2050, climate change will result in higher temperatures and mixed rainfall, harming agricultural production, increasing food costs, and decreasing availability. The effects of climate change will lead to a higher percentage of child malnutrition. Africa is most vulnerable to such changes, since a significant fraction of the population depends on agricultural production for income and fulfilling dietary needs [54]. According to the United Nations International Children’s Emergency Fund (UNICEF) [55], climate change has negatively affected the “Horn of Africa”, whereby a long-lasting drought is affecting Somalia, coastal regions of Kenya, Tanzania, and central-eastern Ethiopia. Some areas have been experiencing below-average rainy seasons for two years, resulting in severe soil moisture deficiency, increased wildfires, and negatively affecting the agricultural sector [56]. There are 7 million, 5 million, and 4 million people who need humanitarian aid in Ethiopia, South Sudan, and Kenya, respectively [57]. Water scarcity will cause a sharp decline in milk production in Kenya and Ethiopia. The prolonged drought will result in lower food yields, death of livestock, and eventually, a large portion of the population will undergo child malnutrition and food insecurity.

In addition, food insecurity is influenced by pandemics caused by easily transmissible plant pathogens. The upsurge of such viruses may result in losing up to 100% of agricultural produce. When abiotic factors are combined with phytopathogens, food insecurity will increase. However, viral infections can be beneficial to the host plant. Some RNA viruses that can alleviate the adverse effects of environmental factors include the brome mosaic virus (BMV), cucumber mosaic virus (CMV), tobacco mosaic virus (TMV), and tobacco rattle virus (TRV). The plants infected with these viruses have a tolerance to drought [58]. There are a few benefits elucidated for TYLCV infection. TYLCV has developed mechanisms that ensure the plant survives more extended periods so that the virus can live inside the host if possible. The TYLCV uses heat shock proteins (HSPs) to prolong infection and ensure the survival of the plants [59].

In cases where plants are exposed to environmental factors, there is an increase in the expression of HSPs. HSPs are chaperones that aid in protein folding, unfolding, and transport. They also partake in degrading and removing non-native proteins [60,61,62]. In the plant cell, HSPs are found in the cytoplasm, nucleus, and cell organelles, such as the mitochondria, chloroplast, and endoplasmic reticulum. During distress, the expression of normal plant proteins decreases, and there is an increase in the expression of stress-related proteins such as HSPs. HSPs are controlled by transcription factors called heat shock factors (HSFs). In plants, HSFs positively regulate plant tolerance to anoxia, osmotic, and oxidative stress [63]. In tomatoes, HSFs are considered master regulators of signal perception, transduction, and control of stress-responsive genes [64,65]. Tomato cultures subjected to prolonged heat stress have an accumulation of HFSA2 [65]. Tomato plants exposed to high temperatures and infected with TYLCV were able to mitigate the heat response by downregulating HSFA2 and HSFB1, while there was an upregulation of HSF-related genes Hsp17, APX1, APX2, and Hsp90 [7]. All TYLCV viral proteins can interact with HSFA2; therefore, it was proposed that HSFA2 be captured by viral proteins of TYLCV, thus inhibiting its translocation into the nuclei [7]. Consequently, this downregulates transcriptional activation of heat stress-response genes [66]. Similarly, drought stress was also mitigated by infection with TYLCV, whereby transgenic plants expressing TYLCV C4 proteins were able to withstand water deficiency better than transgenic plants expressing other complementary sense proteins (C1, C2, and C3) and V2 virion sense protein [67]. Abscisic acid (ABA) is the crucial hormone that regulates drought responses without geminivirus infection; however, during TYLCV infection, drought tolerance was conferred independently of the ABA mechanism. During TYLCV infection, drought tolerance involves HSPs and HSFs [67].

Geminiviruses can be used in biotechnology using CRISPR/Cas systems, ZFNs, and TALENS. Using geminiviruses as vectors permits bypassing the required additional segregation step to remove the coding sequence of the modification tools from transgenic plants because the introduction of geminivirus replicons to plants can be done transiently, similar to a viral infection event [68]. Utilizing geminivirus replicons (GVRs) as vectors for gene manipulation allows for the expression of large modification nucleases, the expression of noncoding single guide RNAs (sgRNA), and provides a high copy number of the homologous DNA repair templates. Geminiviruses have been used in the CRISPR/Cas system, transcription activator-like effector nucleases (TALENS), and zinc-finger nucleases (ZFNs) [69].

Gene editing is a biotechnological group of techniques that can change the DNA of a living organism either by deleting, inserting, modifying, or replacing DNA with a new insert. ZFNs, TALENs, and CRISPR/Cas systems use designer sequence-specific nucleases (SSN) to generate double-strand breaks (DSBs) on the targeted DNA sites. The DBSs are repaired by the host DNA repair systems using nonhomologous end-joining (NHEJ) or homology-directed repair (HDR). During NHEJ, the DNA fragments are joined through enzymatic processes without exogenous homologous DNA. Although this repair mechanism is active in all cell cycle phases, it is error-prone. It may result in the creation of ‘indels’ at the cleavage site, leading to the generation of frameshift mutations or premature stop codons. The HDR mechanism requires a donor repair template consisting of the sequence to be inserted. This DNA repair method allows for the precise insertion or replacement of the gene of interest at the cleavage site. HDR is active in the late S phase and G2 phase of the cell cycle. Manipulation of the geminivirus genome can increase the HDR occurring rather than NHEJ. GVRs act as delivery systems for plants’ recombinant DNA and overexpression of protein. GVRs are simplified systems that require two components of the geminivirus genome: the viral Rep and the conserved sequence recognized by the Rep protein during replication, intergenic region, and common region for monopartite and bipartite geminivirus, respectively. These replicons exclusively depend on the activity of the geminiviral Rep protein. Therefore, the limitations imposed by other viral proteins, such as the independent function of the viral movement protein, are waived.

## 4. CRISPR/Cas Protein Gene Modification

The CRISPR/Cas system is native to bacteria and archaea. It provides these microbes with adaptive immunity against plasmids and viruses. The Cas proteins and guide RNA accumulate, detect, and destroy invading nucleic acids during such circumstances. Doudna and Charpentier found that this could be used to edit any desired DNA by providing the correct template [70]. The CRISPR/Cas system is a gene editing tool used in biotechnology to modify a living system’s DNA more directly. It has been applied in multiple disciplines, such as gene therapy, where medical biotechnology uses the CRISPR/Cas system to replace or repair mutated genes at their native location. This treatment method holds promise to cure human genetic diseases such as β-thalassemia, muscular dystrophy, sickle cell disease, and cystic fibrosis.

In the CRISPR/Cas9 system, CRISPR-associated protein (Cas9) and guide RNA (gRNA) are the two essential components. The gRNA is designed by the CRISPR RNA (crRNA) and transactivating CRISPR RNA (tracrRNA)c to form a single guide RNA (sgRNA). The crRNA serves as complementary RNA that will bind to the specific target DNA sequence, while the tracrRNA functions as a site for binding Cas9 nucleases. Once attached to the tracrRNA, the Cas9 nucleases will recognize and cleave the target DNA site, generating blunt-ended DBS, which are repaired by NHEJ or HDR mechanisms. The CRISPR/Cas system has been adapted to other living systems, such as plants. However, modifications to the system are needed, such as codon optimization and plant-specific promoter selection, to allow for specific edits in plant genomes [71]. Li and colleagues [15] found that the CRISPR/Cas9 system can be adapted to plants by designing an sgRNA:pcoCas9 system that targets the phytoene desaturase that could conduct mutagenesis in *Arabidopsis thaliana* and *Nicotiana benthamiana* plants with HDR and NHEJ repair mechanisms. They also showed that the system could be used as a multiplex to increase targeted mutagenesis and generate deletions to ensure gene knockout. Their sgRNA:pcoCas9 system showed that plant protoplasts can provide functional systems to rapidly evaluate the efficiency of CRISPR/Cas system-mediated gene editing at a specific genomic locus [15].

Current methods of delivery of the CRISPR/Cas9 complex into cells can be grouped into physical (e.g., microinjection, hydrodynamic injection, electroporation), chemical (e.g., lipid and polymer-based nanoparticles), biolistic, and *Agrobacterium* and viral vectors (retroviral vectors, adenoviral vector, adeno-associated viruses, and lentivirus vectors). Viral vectors are used because they have higher efficiency when compared to physical and chemical delivery methods. The efficiency of virus-induced gene silencing was investigated by Xiao and colleagues [72] using cabbage leaf curl virus (CaLCV)- and tobacco rattle virus-based vectors. They found that the CalCV-based vector was cost-effective, as it used *Agrobacterium* for delivery and generated a higher batch efficiency target [72]. The limitation of viral vectors is that viruses have limited cloning capacity; hence, cloning a large protein such as Cas9 becomes a problem. Utilizing geminiviruses for the CRISPR/Cas9 system to deliver necessary gene modifications was investigated by Baltes and co-workers [69], where the deletion of cell-to-cell movement and retaining elements necessary for RCR in the bean yellow dwarf virus (BeYDV) permitted the co-delivery of sequence-specific nucleases and repair templates, increasing replicon amplification. Integration of CRISPR/sgRNA within the geminivirus genome resulted in targeted mutagenesis of the endogenous acetolactate synthase gene.

In genome editing, NHEJ is favored more frequently than HDR because delivering an adequate copy number of donor repair templates into the cell is challenging. To increase the frequency of HDR occurring within the cell, scientists have looked at using GVR to deliver and create a higher copy number of donor repair templates. Kim and co-workers [73] replaced the exon of *Oryza sativa* (rice) with that of lycopene epsilon-cyclase (LycE). Although they achieved a low frequency for the HDR mechanism, the knock-in of LycE increased the total carotenoid content and reduced reactive oxygen species under salt stress conditions. Dahan-Meir and colleagues [74] targeted the carotenoid biosynthesis pathway for gene editing because gene changes will result in phenotypical changes. Their investigation targeted the carotenoid isomerase (CRTISO) and phytoene synthase gene (PSY1) and had no resistance genes in their construct, except for kanamycin, for the transformation stage. This was to create a GVR that would be more amenable for agricultural practices where there would be no need to add exogenous selectable or reporter markers. Dahan-Meir and co-workers’ [74] study resulted in targeted phenotypically glossy and orange mutants, whereas the tomatoes with the repaired CRTISO gene resulted in a matte red fruit. Reasonable herbicide tolerance was achieved by Wang and colleagues [75], where an all-in-one vector construct was used to target the 5-enolpyruvylshikimate-3-phosphate (EPSPS) gene. The achieved gene editing efficiency was 10% to 18% using a Cy4-based CRISPR/Cas9 system. GVR can increase the chances of HDR and the number of donor repair template (DRT) copies [73].

In some cases, mutagenesis and gene targeting (GT) can be attained by stably incorporating GT reagents into the plant genome (*in planta* gene modification) [76,77]. All cells in transgenic plants contain SSN expression cassettes and repair templates. Hence, the desired targeted change could be passed on to the following germline. This would allow for the development of seeds with targeted DNA sequence modification [16]. This may prompt the placement of regulation by the government, thereby increasing the cost of deregulating varieties of crops for commercialization. Gil-Humanes and co-workers [16] used a wheat dwarf virus (WDV) for GT on the hexaploid wheat plants using a Cas9 promoter and ZmUbi promoter to increase the GT frequency by 12-fold over the standard methods.

Genome editing in plants is time-consuming. Therefore, there is a need to develop more efficient and less time-consuming genome editing methods, especially for crops that are difficult to transform and staple foods such as cereals. The challenges of applying the CRISPR/Cas9 system include immunogenicity, lack of a safe and efficient delivery system to the target site, and ethical issues. There are chances of designed sgRNA mismatches to a nontarget DNA site, resulting in nonspecific, unexpected genetic modifications called off-target effects. Such unwanted mutations may lead to harmful effects such as sequence mutations/deletions, sequence rearrangements, and the activation of immune responses. However, these can be mitigated by sgRNA optimization, Cas9 nuclease modification, utilization of other Cas-variants, and anti-CRISPR proteins. The generation of multi-copy numbers using geminivirus is dependent on efficient viral replication following RCR that is coupled with low viral load and no symptoms. The BeYDV-based vector was deconstructed to allow for the insertion of CRISPR reagents whilst still maintaining replication. Deconstruction was conducted by the removal of the CP and MP ORFs [69]. Moreover, the monopartite BeYDV replication relies on SIR, LIR, and Rep and Rep A. The Rep protein binds to the LIR to initiate RCR, and Rep A interacts with plant cell proteins that encourage progression in the S phase [68]. GVRs can then deliver other gene editing reagents such as ZFNs and TALENS (Table 1).

## 5. TALENs and ZFNs

ZFNs were first engineered in 1996 by Chandrasegaran and colleagues. ZFNs and TALENs are similar in architecture; they contain DNA binding and cleavage domains. In TALENs, the DNA binding domain is made of tandem repeats of TALEs like phytopathogenic bacteria from the *Xanthomonas* genus. The TALEs and zinc-fingers are fused to the C-terminal DNA cleaving domain. Their DNA cleavage domain is nonspecific and is adapted from type II restriction endonucleases, FokI, which functions once dimerization occurs. Therefore, a pair of ZFNs or TALENS must bind to the DNA of interest in opposite directions on target nonpalindromic DNA sites, separated by a spacer. Once DSBs are generated, the plant DNA machinery will repair the DBS, resulting in one of the following: gene addition, gene mutagenesis, gene targeting, or gene editing. However, small indels can arise from unfaithful DNA repair during TALEN-mediated gene editing. In some cases, this results in 1 bp indels larger than those created by the CRISPR/Cas9 system [85].

Like the CRISPR/Cas system, TALENs and ZFNs can be delivered in four categories. The delivery method chosen is dependent on the target organism and aim. The most common form of plant delivery is via agrobacterium-mediated callus, embryo, and leaf explant transformation. This delivery method, theoretically coupled with GVR, should increase the genome efficiency of TALENs because of the large number of TALEN reagents generated during geminiviral replication. Kimm and colleagues [85] found that TALENs can be delivered using BeYDV-based replicons. Co-delivery of the reagents did not increase the efficiency of the genome in potatoes. Moreover, the use of GVR did not improve the frequency of NHEJ. Butler and co-workers [81] also found that GVR-delivering TALEN reagents could induce targeted NHEJ mutations in transformed events. However, the strategy did not increase the efficiency of TALEN-mediated gene editing. ZFNs have not been popularly used utilizing geminivirus replicons for delivery. However, they have been used to inhibit/increase resistance/tolerance to geminiviruses.

## 6. Geminivirus Resistance in Plants Using CRISPR/Cas9, TALENs, and ZFNs Technology

Zinc-finger technology has allowed plants to have enhanced resistance to the beet severe curly top virus (BSCTV) and TYLCV. Instead of cleavage of the viral DNA, artificial zinc-finger proteins (AZPs) are used [86]. AZPs are designed to bind six-AZPs to a 19 bp DNA, including the Rep binding site of the geminivirus. The AZPs are constructed to have a higher binding affinity than the Rep protein. This is to ensure that the AZPs will inhibit the initiation of replication. Takenaka and co-workers [87] designed transgenic *A. thaliana* plants expressing AZPs with binding affinity to the BSCTV. The resistance to the BSCTV was hereditable to the generation preceding T_1_ transgenic plants. No in vivo work has been reported for the TYLCV. However, several in vitro studies [88,89,90] showed success. The advantage of this approach is the lack or reduced risk of the rapid development and occurrence of virus mutants resistant to inhibiting the viral protein binding to the AZPs. To overcome this type of resistance, two mechanisms must occur: (i) rapid evolution of the virus DNA genome to inactivate the AZPs and (ii) simultaneous mutation of the viral replication proteins and DNA sequences resulting in the overall viral genome mutation. Both events are unlikely, as no gene encoding an enzyme capable of “turning off” the activity of AZPs by chemical modification (e.g., phosphorylation) has been identified, and the possibility of a synchronized double mutation event occurring is relatively low [87].

Due to difficulty and cost, TALEN technology has yet to be used to increase plant resistance using plant biotechnology. However, TALEs, which lack the nuclease domain, have been developed to combat begomoviruses. Transgenic *N. benthamiana* plants expressing two artificial TALE proteins, the conserved hairpin and Rep protein of the tobacco curly shoot virus (TbCSV), displayed partial resistance to three begomoviruses: TbCSV, tomato leaf curl Yunnan virus (TYLCYnV), and tomato yellow leaf curl China virus (TYLCCNV) alone and together with its associated beta satellite [91]. A level of resistance was achieved in *N. benthamiana* plants under co-infection with the cotton leaf curl virus (CLCuV) and the cotton leaf curl Kokhran virus (CLCuKV) [92]. Resistance was characterized by a delay in the development of symptoms and a reduced virus titer in systemic leaves.

The CRISPR/Cas 9 machinery can be integrated into the plant’s genome to generate transgenic plant lines that express either the sgRNA or the Cas9 protein. Tashkandi and co-workers [93] created a Cas9 endonuclease-stably expressing sgRNA molecule that was introduced into *N. benthamiana*. The sgRNA targeted the CP, the RCRII motif of the Rep, and the IR of several TYLCV coding and noncoding regions. All sgRNAs could disrupt the TYLCV genome sequence, but focusing on the stem-loop invariant region found in the IR led to a more pronounced decrease in viral replication and accumulation. BCTV and the merrenia mosaic virus (MeMV), which have an identical stem-loop sequence in the IR, were examined using the same technique. The outcomes showed that mixed infection immunity could be generated using sgRNA specific for conserved multiple strains by attenuating symptoms for both viruses. Transgenic *N. benthamiana* and tomato “Money marker” plants expressing the CRISPR/Cas reagents targeting the TYLCV CP and Rep were used by Rajabu and colleagues [94] to show increased resistance to TYLCV. They further demonstrated the CRISPR/Cas system targets the ssDNA of TYLCV, thus hindering viral replication by targeting and cleaving the essential ORFs for viral replication. The mechanism of the CRISPR/Cas system in geminiviral replication is highly specific to the virus to be targeted. It depends on an excellent sgRNA to bind to the region of interest and which region of interest in the genome. However, multiple virus resistance can be achieved if the target region of the genome is conserved between viruses.

Introgression breeding for resistance has been practiced for many years for agriculturally important crops. Tomatoes are one of the most infected crops by geminiviruses. They are susceptible to more than 90 begomoviruses [95]. TYLCV is among the most damaging and threatening viral diseases for tomato production worldwide. Six genes, Ty1-Ty6, have been identified as resistance genes against the TYLCV. These genes have been characterized to encode an RNA-dependent RNA polymerase, a nucleotide-binding-leucine-rich protein, and a messenger RNA (mRNA) surveillance factor, Pelota (*Pelo*) [96,97,98]. The Ty-5 gene confers broad resistance to various begomoviruses, including a co-infection of TYLCV, tomato yellow leaf curl China beta satellite, and beet curly top virus (BCTV) [99]. The co-infection of the TYLCV compromises the resistance plants gained by incorporating the TY1 gene with a beta satellite [100]. However, modern technology has increased plant resistance by limiting the time required to achieve it. The CRISPR/Cas system has been used to target the genome of geminiviruses to inhibit plant replication, resulting in resistant plants characterized by limited virus-induced phenotypical changes and a reduced viral load [101]. The SlPelo gene is a critical factor in TYLCV-mediated disease development. Therefore, the knockdown of this gene in a Ty-5-absent tomato plant, using CRISPR/Cas technology, resulted in reduced TYLCV DNA and a TYLCV-resistant phenotype [102].

Standard methods used to treat geminivirus rely primarily on managing the insect vector with high-toxicity insecticides. However, the use of insecticides/pesticides results in concomitant problems of environmental concerns, low cost–benefit ratio, and pesticide-resistant variant pathogen development. Another treatment method, integrated pest management (IPM), was introduced to decrease issues centered around geminivirus treatment. IPM is a long-term preventive measure against pests that involves combinational techniques such as biological control, habitat manipulation, modification of cultural practices, and resistant plant varieties. Such plants include tomatoes, cotton (*Gossypium hirsutum)*, and cassava (*Manihot esculenta*). In this strategy, the pesticides are used according to guidelines. They are selected and applied to minimize the risk to human health and benefit plants, nontargeted organisms, and the environment. The biotechnology techniques discussed in this article have been used to generate geminivirus-resistant plants.

## 7. Enhancing Plant Resistance: A CRISPR-Cas or TALENs/ZFNs Approach to Overexpressing Salicylic Acid (SA) and Jasmonic Acid (JA)

Phytohormones are small endogenous, low-molecular signaling molecules produced naturally by plants [103]. They regulate natural processes such as plant growth and development, flowering, and senescence. Furthermore, they are released during defense response against biotic and abiotic stressors. Various plant hormones have been identified to be involved in geminiviral infection, such as ethylene, salicylic acid (SA), jasmonic acid (JA), gibberellic acid, auxin, and cytokinin. During a geminivirus invasion, there is an increased concentration of SA in the plant to establish local and systemic acquired resistance by producing pathogenesis-related proteins (PR). Plant resistance increased in TYLCV-infected tomato plants through the induction of SA-responsive PR genes (SlPR1, SlPR2, and SlPR5) and ROS scavenging enzymes (SlSOD, SlPOD, and SlCAT2) [104]. SA-induced plant resistance can be achieved by priming with SA or its derivatives, infection with a microorganism, or using biotechnology techniques.

The constitutive expression of PR genes 1 (CPR1) acts upstream of the SA pathway. It acts as a repressor of pathogen signaling. It mediates the turnover of R-proteins such as SNC1 and RPS2, thus counteracting the induction of effector-triggered immunity (ETI). *A. thaliana* plants harboring a cpr1 mutation constitutively expressed the PR1 and PR5 genes. This increased the plant’s resistance to the fungal pathogen *Peronospora parasitica* NoCO2 and the bacterial pathogen *Pseudomonas syringae* pv maculicola ES4326 [104]. Similar results were attained by Ascencio-Ibáñez and colleagues [105], where cpr1 mutant plants infected with CaLCV showed delayed symptom display for up to 25 days and increased transcript levels of SA-responsive PR genes. Similar results have been achieved for BSCTV. Chen and co-workers [106] screened mutants resistant to BSCTV infection and identified one mutant named less susceptible to BSCTV 1 (LSB1). The lsb1 mutation impaired DNA replication and decreased the BSCTV infectivity, resulting in an increase in the expression of Glutamine Dumper 3 (GDU3). GDU3 then concomitantly increased the expression of several SA pathway components such as accelerated cell death 6 (ACD6), pathogen and circadian controlled 1 (PCC1), cell wall-associated kinase 1 (WAK1), and PR5. Geminiviral pathogenesis is negatively affected by SA accumulation, which is noticeable by the reduction in viral titer. Furthermore, plant resistance to TYLCV increases after exogenous administration with SA and JA [107]. During geminivirus infection, genes are upregulated in the SA pathway, and genes involved in the JA pathway are differentially regulated [103,108].

**Figure 3:**
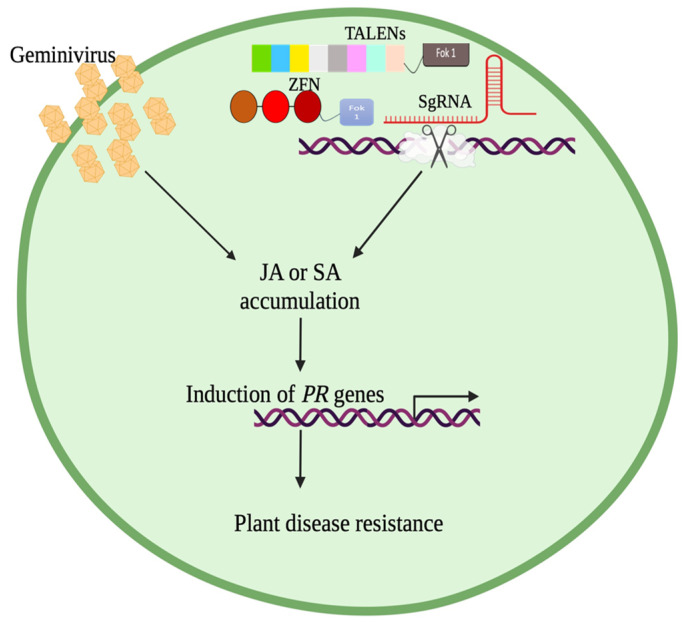
Geminivirus infection causes an increase in jasmonic acid (JA) and salicylic acid (SA), and this accumulation induces a pathogenesis-related (PR) gene, improving plant disease resistance. Gene editing techniques (ZFN, TALENs, and CRISPR/Cas9) used to manipulate plant DNA may also increase plant resistance through JA or SA accumulation.

To maintain a homeostatic environment, plants need to regulate the effects of one physiological change in their environment. For instance, the accumulation of SA caused by microbial invasion negatively affects the synthesis of JA. This antagonistic interplay is displayed in the SA/JA crosstalk. During transmission of the virus by the whitefly, SA synthesis is induced [109,110], which, upon feeding on the plant phloem, the whitefly releases a salivary effector molecule, Bt56, which partially inactivates NTH202, a KNOX transcription factor, resulting in the accumulation of SA. This is to increase the performance of whiteflies by decreasing the JA-mediated anti-herbivore immune response [110]. JA increases plants’ resistance to herbivores by regulating the synthesis of harmful defensive proteins. For example, terpenoids improve results in resistance against whiteflies. The overexpression of 35s-pro systemin in plants results in JA-mediated resistance. Tomato plants overexpressing 35s-protein and infected with TYLCV decrease the performance of the whitefly. TYLCV did not repress JA biosynthetic genes (AOC, AOS, OPR3, and LOXD); however, the transcript levels of PI-II, a JA-regulated defense gene, were down-regulated [111]. Once inside the host, begomoviral proteins interact with the plant’s machinery to increase the plant’s susceptibility; for example, βC1 protein interacts with MY2, which represses JA-mediated responses [112]. The JA-mediated defense response can then decrease viral titer, leading to an observation of milder symptoms [113]. JA can be accumulated by using mutant plants to increase biotic resistance. The patatin-like protein 2 (plp2) expression increases during CaLCV infection [114], but the mutation of the plp2 gene results in the accumulation of JA [110]. Patatin-related phospholipase A (pPLAII-alpha) hydrolyzes membrane glycerolipid to produce lysolipids and free fatty acids, while gene knockout increases the basal levels of JA, methyl-JA, and other intermediates such as linolenic acid, 13-hydroperoxylinolenic acid (13-HPOT), 12-oxophytodienoic acid (OPDA), and increased expression of JA biosynthetic genes [115]. GVRs can achieve increased resistance in plants by maneuvering plant phytohormone pathways. Increasing the plant’s SA or JA concentration will increase the plant’s resistance, and potentially yield improved crop quality and quantity (Figure 3).

## 8. Biotechnology for Sustainable Agriculture in Africa

Food insecurity is a major problem in Africa. Two factors that negatively impact food security are population growth and climate change. Between 2015 and 2100, there is predicted to be a five-fold increase in population for some of Africa’s poorest countries: Somalia, Niger, Tanzania, Malawi, Zambia, and Uganda [116]. Such an increase in population could make achieving Sustainable Developmental Goals (SDGS) like “Goal 2: end hunger, achieve food security and improved nutrition and promote sustainable agriculture” more challenging. This is further exacerbated by climate change. Changes in rainfall patterns and temperature affect crop yield, plant pests and diseases, as well as the availability of water. Some of Africa’s stable foods, such as wheat, sorghum, millet, and maize yield are projected to be negatively impacted by climate change by 2050 [117]. Therefore, generating weather- and pathogen-resistant plants through genetic modification would help elevate African food insecurity.

Recently, African scientists have partnered with institutions to propel plant genome editing. For instance, the International Institute of Tropical Agriculture is genetically editing bananas for resistance against bacterial and viral diseases [118]. Corteva Agriscience has partnered with Ethiopian and Kenyan scientists to develop genetically modified sorghum to manage the intractable parasitic weed *Striga* [119]. *Striga*-resistant genetically modified sorghum plants have been developed using CRISPR. Gobena et al. [120] identified that mutation of the LOW GERMINATION STIMULANT 1 (LGS1) locus reduced the germination stimulant of *Striga*. The development of *SgLGS1* silenced sorghum plants using the CRISPR/Cas system was conducted by Kenyatta University, Kenya. Similarly, knockout of genes that encode carotenoid cleavage dioxygenase was achieved using CRISPR and decreased the germination of Striga, albeit the sorghum crop yield decreased [121].

Cassava is a staple food in most African homes. The majority of the cassava produced in Africa is by smallholders, and contributes to 60% of the global production of cassava [122]. However, its production is limited by phytopathogens such as ACMV, cassava brown streak virus (CBSV), and *Xanthomonas axonopodis* pv. *manihotis* (Xam) causing cassava bacterial blight (CBB). The gene function of Ubiquitin E3 Ligase in response was investigated in cassava plants by the transient expression of the CRISPR/Cas system [123]. Although successful in the characterization of genes, the authors of the newly developed protocol could not regenerate plantlets from the cassava protoplasts [123]. The global impact of cassava mosaic disease can total a loss of more than USD 1 billion [124]. CRISPR/Cas9 technology, targeting the viral AC2 gene, was employed to generate cassava mosaic disease-resistant plants [125]. In the study, transgenic cassava plants failed to confer resistance to the plant disease. Furthermore, the manipulated viral genome developed resistance to the CRISPR/Cas9 cleave by generating a conserved single nucleotide mutation. Gomez et al. [126] developed a CRISPR/Cas9 system that targeted the novel cap-binding protein 1 (nCBP-1) gene of the cassava plant. The ncbp-1/ncbp-2 mutant plants developed a tolerance to CBSV that was characterized by reduced viral load, decreased disease severity, as well as delayed and attenuated CBSV symptoms [126]. The resistance to CBB was achieved by mutation of the promoter of the *MeSWEET10a* gene, and CRISPR-induced mutation did not cause any morphological changes to the mutated cassava plants [127]. Other modifications to the cassava genome include mutagenesis in the CYP79D1 gene, reduced levels of cyanide and Protein targeting to starch (*PTST1*) or Granule bound starch synthase (*GBSS*)—targeted gene modification lowered amylose content [128,129].

Increased knowledge and improvement of biotechnology have influenced the use of the CRISPR system to enhance resistance to various factors in numerous plants; seed shattering was reduced in *Oryza glaberrima* (African rice) by the knockout of Seed Shattering 11 gene, multigenic foxtail genome mutation in millets created an herbicide-tolerant plant, and knockdown of the maize poly(ADP-ribose) polymerase (PARP) 2 gene exhibited tolerance to oxidative stress [130,131,132]. Research on the use of TALENs and ZFNs for crop improvement is limited in the African content. The development of biotic- and abiotic-resistant or tolerant crop varieties has the potential to alleviate food insecurity. Using biotechnology tools can improve gene targets, as techniques like ZFN, TALENs, and CRISPR/Cas9 systems permit precise gene editing. Furthermore, gene editing can be used to create biofortified foods resulting in crops with improved nutritional content. The use of GVRs for genome manipulation have yet to be reported in Africa.

## 9. Conclusions

Geminiviruses cause a loss in the production of agriculturally important crops. However, plant infection with these viruses results in increased plant tolerance to abiotic stressors such as heat and water scarcity. Additionally, they can be used in agricultural biotechnology to generate GVR and edit crop genomes for the plant’s benefit. Gene manipulation, such as single nucleotide mutation or whole gene knockout, can be achieved in plants faster with increased precision utilizing techniques such as the CRISPR/Cas 9 system, TALENs, and ZFNs. These techniques can be used to edit the genome of plants to manipulate phytohormone pathways such as SA and JA. Such gene editing can use GVRS and increase the plant’s resistance to biotic and abiotic factors. Increasing the plant’s resistance to such stressors could aid in the fight against food insecurity in Africa.

## Figures and Tables

**Table 1:** Geminivirus-based replicons used to achieve gene editing in plants.

Virus Used	Targeted Gene	Plant	Year	Ref
Bean yellow dwarf virus (BeYDV) ^a^	Carotenoid isomerase (*CRTISO*) Phytoene synthase 1 (*PSY1*)	Tomato	2018	[74]
BeYDV ^a^	*GhCLA1*	*Gossypium hirsutum* (Upland cotton)	2022	[71]
BeYDV ^a^	*SlHKT1;2*	Tomato	2020	[78]
BeYDV ^a^	Lycopene epsilon-cyclase (*LcyE*)	Rice	2022	[73]
BeYDV ^a^	Anthocyanin mutant 1 (*ANT1*)	Tomato	2015	[79]
BeYDV ^a^	Auxin induced in root culture 12 (*VviAIR12*) Sugar will eventually be exported transporter 4 (*VvSWEET4*) Lesion initiation 2 (*VviLIN2*) Dimerization partner-E2F-like 1 (*VviDEL1*)	*Vitis vinifera* L. (Grape)	2021	[80]
BeYDV ^a^	Acetolactate synthase 1 (*ALC1*)	*Solanum tuberosum* L. (potato)	2016	[81]
Cabbage leaf curl virus (CaLCV) ^b^	Phytoene desaturase 3 (*NbPDS3*) *NbIspH*	*N. Benthamiana*	2015	[82]
BeYDV ^a^	Solyc06g074350 Solyc02g085500 Solyc02g090730 Solyc11g071810 Solyc06g074240 Solyc02g07739 Ubiquitin 5-enolpyruvylshikimate-3-phosphate synthase (*EPSPS*) Mildew locus O [*TaMLO*] Hordeum vulgare; *HvMLO*	Tomato	2017	[83]




		Wheat		





		Barley		
BeYDV ^a^	Vacuolar invertase 1 (*StvacINV1*) Beta-amylase 1 (*StBAM1*) Polyphenol oxidase 1 and 2 (*StPPO1* and *StPPO2*)	Potato	2021	[84]
BeYDV ^a^	Enolpyruvylshikimate-3-phosphate synthase (*EPSPS*)	*Brassica napus* L. (Rapeseed)	2021	[75]
Wheat dwarf virus (WDV) ^a^	Mildew locus O (*MLO*) *EPSPS*	*Triticum* sp. (wheat)	2017	[16]

^a^ Mastrevirus genus, ^b^ Begomovirus genus.

## Data Availability

Not applicable.
